# Longitudinal effects of early psychosocial deprivation on macaque executive function: Evidence from computational modelling

**DOI:** 10.1098/rspb.2022.1993

**Published:** 2023-04-12

**Authors:** Alice Massera, James J. Bonaiuto, Marine Gautier-Martins, Sara Costa, Holly Rayson, Pier Francesco Ferrari

**Affiliations:** ^1^ Institut des Sciences Cognitives – Marc Jeannerod, CNRS UMR5229, Bron 69500, France; ^2^ Université Claude Bernard Lyon 1, Université de Lyon, 69100, France; ^3^ Unit of Neuroscience, Department of Medicine and Surgery, University of Parma, Parma 43125, Italy

**Keywords:** executive function, macaque, computational modelling, working memory, inhibitory control, early psychosocial deprivation

## Abstract

Executive function (EF) describes a group of cognitive processes underlying the organization and control of goal-directed behaviour. Environmental experience appears to play a crucial role in EF development, with early psychosocial deprivation often linked to EF impairment. However, many questions remain concerning the developmental trajectories of EF after exposure to deprivation, especially concerning specific mechanisms. Accordingly, using an ‘A-not-B’ paradigm and a macaque model of early psychosocial deprivation, we investigated how early deprivation influences EF development longitudinally from adolescence into early adulthood. The contribution of working memory and inhibitory control mechanisms were examined specifically via the fitting of a computational model of decision making to the choice behaviour of each individual. As predicted, peer-reared animals (i.e. those exposed to early psychosocial deprivation) performed worse than mother-reared animals across time, with the fitted model parameters yielding novel insights into the functional decomposition of group-level EF differences underlying task performance. Results indicated differential trajectories of inhibitory control and working memory development in the two groups. Such findings not only extend our knowledge of how early deprivation influences EF longitudinally, but also provide support for the utility of computational modelling to elucidate specific mechanisms linking early psychosocial deprivation to long-term poor outcomes.

## 1. Introduction

Executive function (EF) refers to a group of cognitive processes that underlie the organization and control of goal-directed behaviour. One prominent theory defines EF as a construct comprising a number of interrelated, but distinct, components [1]. These include working memory, inhibitory control and cognitive flexibility, with some common underlying processes connecting them [2]. EF develops rapidly between birth and 2 years of age, with steady development then continuing into early adulthood [3,4]. Such protracted development suggests continued plasticity of EF, as does the extended developmental trajectory of neural networks supporting EF (e.g. fronto-parietal network) [3,5,6]. Critically, individual differences in childhood EF are related to later social competence, academic performance and occupational functioning [7–9], as well as risk for interpersonal problems and risky behaviour during adolescence and adulthood [10,11]. EF deficits are also characteristic of a number of neurodevelopmental disorders, including attention deficit/hyperactivity disorder (ADHD) and autism spectrum disorders [12–14]. However, despite the clear importance of EF for healthy development [15], many questions remain concerning the factors underlying different developmental trajectories of EF and thus the individuals most at risk for poor outcomes.

Evidence is growing for early environmental experience playing an important role in EF development. Indeed, early social adversity has been consistently linked to EF impairments in childhood (e.g. [16–18]), with early psychosocial deprivation, a specific form of early social adversity involving removal of an infant from their primary caregivers, having a particularly profound effect on EF components such as working memory and inhibitory control [19–22]. Relevantly, increased risk for ADHD is also associated with early psychosocial deprivation [23,24], as is risk for symptoms such as disinhibited social engagement and repetitive and stereotyped behaviour [25]. A lack of complexity and socio-cognitive stimulation in the early social environment, along with increased levels of stress, could explain why early psychosocial deprivation has such a significant impact on EF. For example, this can lead to excessive and less selective pruning of synapses in the cortex, including prefrontal regions implicated in higher cognitive abilities [26]. In support of this, deprivation has regularly been associated with a generalized reduction of grey matter in prefrontal, parietal and temporal cortical regions [27].

The few longitudinal studies conducted on this topic suggest that poor EF associated with early psychosocial deprivation in early life can persist into adolescence and adulthood [21,24,28,29], but as most studies only evaluated EF at one time-point [19,20,30,31], many questions about long-lasting effects on EF and the neural mechanisms underlying this relationship across time remain unanswered. Longitudinal research is now essential for enhancing our understanding of how various aspects of cognition develop in terms of both typical and atypical trajectories. The tracking of EF across adolescence into adulthood will be a particularly key period of transition for future examination in studies of psychosocial deprivation. Adolescence is a period of heightened brain plasticity defined by the significant development of prefrontal cortex [32,33], as well as important refinements of EF [6]. As such, adolescence may be a particularly useful target for interventions aimed at preventing or ameliorating adverse effects of early deprivation on cognitive development.

The use of a non-human primate model such as rhesus macaques (*Macaca mulatta*) could be very helpful to address outstanding questions about the effects of early psychosocial deprivation on EF across development. Macaque monkeys are one of the closest species to humans in terms of genetics, physiology and behaviour, and have an extended period of development comprising distinct infant, juvenile (pre-adolescent and adolescent) and adult stages. Like humans, their early social environment predominantly consists of mother–infant interactions. Notably, the use of a macaque model can help address some limitations defining developmental studies with humans (e.g. lack of control over the early environment, very long developmental time frames and difficulty in tracking neural mechanisms underlying development). Although limited, research focused on EF development in macaques suggests that it parallels that in humans [34–37], and in adult macaques, EF relies on the same cortical networks [38,39]. Limited research focused on early psychosocial deprivation effects on cognition in infant macaques have produced mixed findings (e.g. [40,41]), with research comparing deprived versus non-deprived animals especially rare. Discrepancies in findings concerning effects of early psychosocial deprivation on cognitive ability may stem from differences in age at assessment and use of different measures at different time-points, and no studies thus far have focused on EF specifically.

A classic paradigm used to investigate EF in human infants and young children is the ‘A-not-B’ task [42–44], which is thought to measure two EF components: inhibitory control and working memory [45–47]. Modified versions of the ‘A-not-B’ task have also been used to investigate EF in trained adult macaques. Macaque behaviour during the task is comparable to human performance [45,48]. No previous research has used an ‘A-not-B’ task to look at macaque EF development or effects of early psychosocial deprivation, though a version such as that used with human infants could offer a valuable way to test EF without removal of the animals from their home enclosure and extensive training. A data collection approach that requires these two elements is starting to be adopted by other researchers [41,49], who argue that it enables a more direct comparison of cognitive development in macaques exposed to early psychosocial deprivation versus non-exposed macaques.

Although the ‘A-not-B’ task is assumed to involve both working memory and inhibitory control mechanisms, it has been recently argued that these components are difficult to disentangle using classic measures of response accuracy according to condition [50,51]. Developing tasks that do not rely on instruction in order to assess specific EF components in infancy and early childhood [50,51] is very challenging, but another option to help isolate mechanisms underlying behaviour in the ‘A-not-B’ task is to use computational modelling. Specifically, using a computational model of decision making to functionally decompose choice behaviour into working memory and inhibitory control mechanisms would enable both examination of these specific EF components, as well as a way to evaluate how well previously used ‘A-not-B’ performance measures actually assess these components.

Accordingly, the current study was designed to investigate the longitudinal effects of early psychosocial deprivation on EF in rhesus macaques using a computational modelling approach. We assessed EF in a rare sample of macaques comprised two groups that differed in exposure to early psychosocial deprivation; one mother-reared and one peer-reared (i.e. exposed to early psychosocial deprivation; separated from their mothers and other adults at birth and raised in a nursery of peers by human caretakers); at two time-points corresponding to adolescence (3.5 years) and early adulthood (5 years). We used a version of the ‘A-not-B’ paradigm often used with human infants [47], with animals completing the task in a section of their home enclosure. The choice behaviour of each animal at each time-point was fit with a stochastic computational model of decision making based on a weighted sum of exponentially decaying working memory and choice history influences. Choice history corresponds to inhibitory control, the tendency to suppress previous responses or to repeat them. We then compared the fitted model parameters between groups and across time, as well as to previously used performance measures [47,52]. We predicted that peer-reared animals would perform worse than mother-reared animals on the task at both time-points, and that both working memory and inhibitory control would be poorer in the peer-reared group.

## 2. Methods

### (a) Subjects

The sample consisted of 21 rhesus macaque monkeys (*Macaca mulatta*), of which 11 were mother-reared (five female) and 10 peer-reared (six female). Subjects were aged around 3.5 years at the first assessment time-point (mother-reared; *M* = 1368 days, s.d. = 104 days: peer-reared; *M* = 1363 days, s.d. = 101 days) and 5 years at the second assessment time-point (mother-reared; *M* = 1806 days, s.d. = 100 days: peer-reared; *M* = 1809 days, s.d. = 95 days). Subjects were housed at the Institut des Sciences Cognitives Marc Jeannerod, CNRS, during the assessment period in mixed mother- and peer-reared social groups of 5–6 animals (see electronic supplementary material for rearing protocol). Although new instances of maternal separation in monkeys' for research are largely prohibited, it was decided that further study of this particular sample of juvenile animals was the most ethical course of action. Due to the closure of the centre in the USA where animals were born and lived for the first 2 years of life, it was agreed that our team at the ISC-MJ would receive these animals rather than allow them to be euthanized for medical research whereby effects of their early social experiences would not be considered, and consequently would fail to maximize the scientific benefit that could be derived from this existing sample. Every effort has been made to ensure that these animals now live in the most enriching environment possible, including their social environment, and the tasks included in the current study were designed to be as non-invasive as possible. All housing and procedures conformed to current guidelines concerning the care and use of laboratory animals and were approved by the relevant authorities (see 'Ethics'). All reporting here conforms to the recommendations in the ARRIVE Guidelines for Reporting Animal Research.

### (b) ‘A-not-B’ task set-up and procedure

Each subject was temporarily separated into the testing area, which was a section of the home enclosure, an 87 × 100 × 120 cm area with a clear-panelled front. All equipment required for the task was installed before separating the subject. Note, all animals had already been well familiarized with this process of separation into the testing area and with the task equipment.

**Figure 1.**
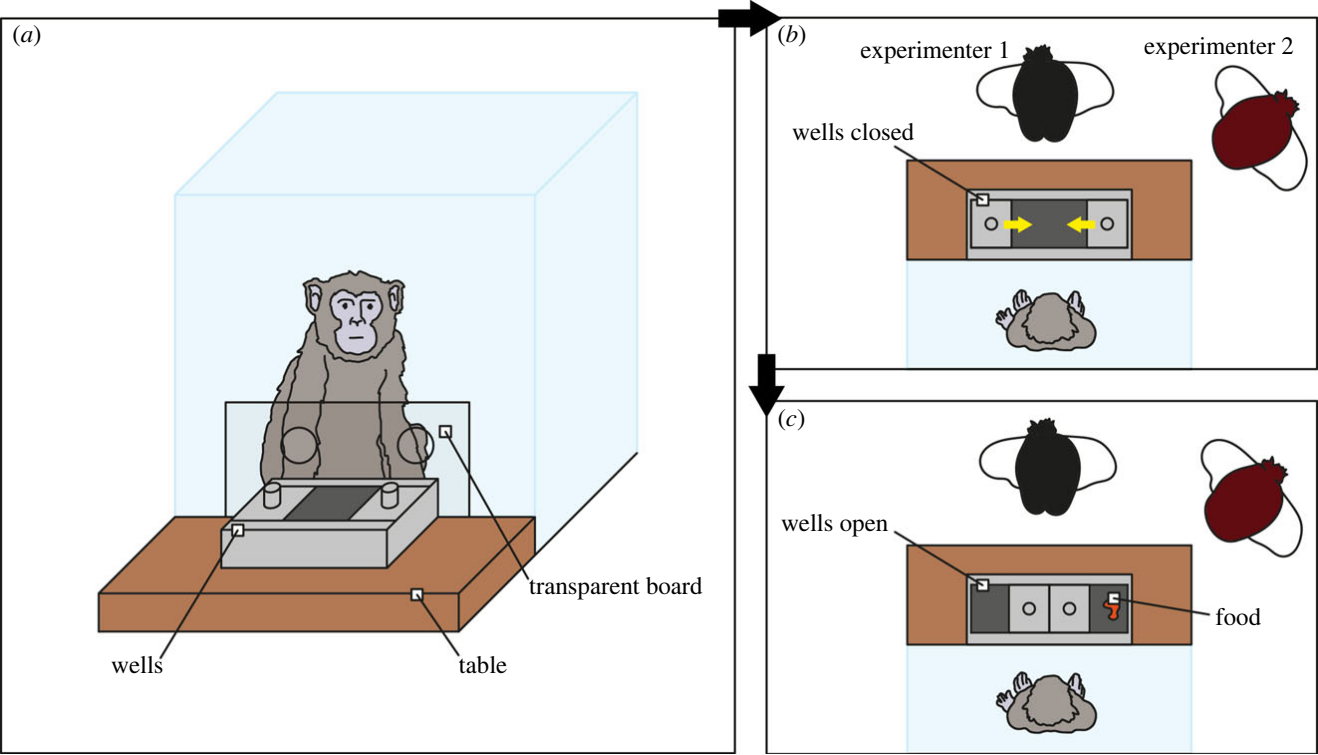
Task set-up. (*a*) A frontal view of the test enclosure. (*b*,*c*) Two views from above: (*b*) the wells are closed with the yellow arrows indicating a sliding mechanism; (*c*) the wells are open and a piece of food is placed in one of them.

The task set-up included two holes in the clear-panelled front of the testing area which enabled the subject to reach objects placed on a table outside the enclosure (figure 1). A transparent board was used to block these holes at times when the subject was not allowed to reach (i.e. during the hiding and delay portions of the task). A box with two wells and two sliding doors to cover the wells was positioned centrally on the table in front of the two holes. The wells were 11.2 cm in diameter and were positioned 18 cm apart. Two experimenters were present for the entire task. Experimenter 1 stood facing the subject, while experimenter 2 stood on one side of the table, with the side counterbalanced across subjects and sessions.

**Figure 2.**
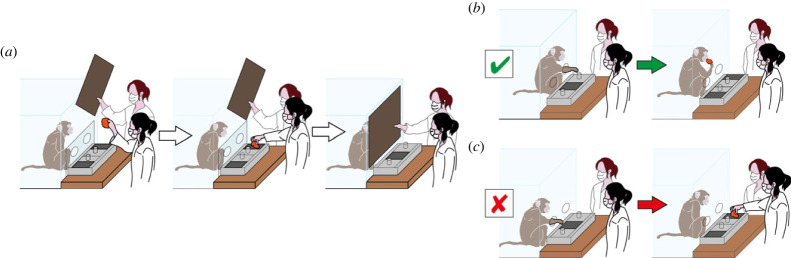
Task procedure. The series of images illustrates the sequence of a single trial. (*a*) Experimenter 1 shows the food to the subject, then places it in the well, and experimenter 2 blocks the vision of the subject during the delay period. (*b*) A ‘correct’ choice, with the subject reaching for the well where the food was hidden and then eating the food. (*c*) An ‘incorrect’ choice, with the subject choosing the well containing no food and experimenter 1 then highlighting where the food was actually hidden.

To begin a trial, experimenter 1 showed a piece of food to the subject in the centre of their visual field (figure 2*a*). Once the subject looked at the food, experimenter 1 placed the food in one of the wells and closed the two sliding doors to cover both wells. The trial only proceeded if the subject looked at the food when it was presented and when it was hidden. As soon as the wells were covered, a delay period started. During this delay, as well as physical access to the wells being blocked by the transparent board, experimenter 2 blocked the subject's visual access to the wells with an opaque screen placed between the box and the enclosure front. At the end of the delay, the opaque screen and the transparent board were removed, allowing the subject to reach for the wells. If the subject reached for the correct well, the experimenter allowed the subject to eat the piece of food (figure 2*b*). If the subject reached for the incorrect well, the experimenter opened the correct well to show the position of the food to the subject (figure 2*c*). If the subject touched both wells, did not respond, or the response was unclear, the trial was repeated. The food was hidden in the same location (left or right well) until the subject reached for the correct well on two consecutive trials. After two consecutive correct trials, experimenter 1 changed locations and hid the food in the other well (i.e. it was a ‘change’ trial). After the change trial, the experimenter then repeated this sequence but on the same side as the preceding change trial. For the first trial of each session, the delay period when visual and physical access to the wells was blocked lasted 2 s, and then depending on the subject's performance, it was increased or decreased in subsequent trials. The delay period was decreased by 2 s if two consecutive trials were incorrect, and was increased by 1 s if two consecutive change trials were correct [47,52]. Each subject completed two testing sessions at each assessment time-point, with a maximum of 25 trials per session (i.e. 50 trials total per assessment time-point). During each session, experimenter 1 live-coded correct and incorrect trials, and animals were also video recoded throughout the session with a camera placed to capture a view of the enclosure and experimenter 1.

### (c) Control task set-up and procedure

At the second assessment time-point, all subjects also completed a control task to verify that results from the original version of our ‘A-not-B’ task were not a consequence of the structure of the task. In the original version, the length of the delay period depended on the performance of the subject, with poorly performing subjects not being tested at higher delays. Moreover, if the subjects were always correct, they could have possibly learned the pattern of the hiding locations (e.g. A, A, B, B, B, A, A, B). Therefore, while the task set-up was the same as in the original ‘A-not-B’ task, the procedure was different. In this new randomized control version of task, the position of the food hiding location (i.e. left or right well) and the length of the delay period were pseudo-randomized across trials. The hiding position could not be the same for more than five consecutive trials, and the delay period was maximum of 5 s. All subjects completed the same number of trials with a specific delay in a specific location (e.g. 3 s, right well). Each subject completed two sessions, with a maximum of 24 trials per session (i.e. 48 trials total). With this version of the task, then, all animals were tested equally on all the different possible delays (i.e. from a 0 s delay to a 5 s delay).

### (d) Video coding

A number of parameters were coded offline from the video recordings made of each subject during the testing sessions, including the actual length of delay periods (which may have varied slightly from the intended delay period) in seconds and the inter-trial interval (ITI) lengths in seconds (see electronic supplementary material).

### (e) Computational model

**Figure 3.**
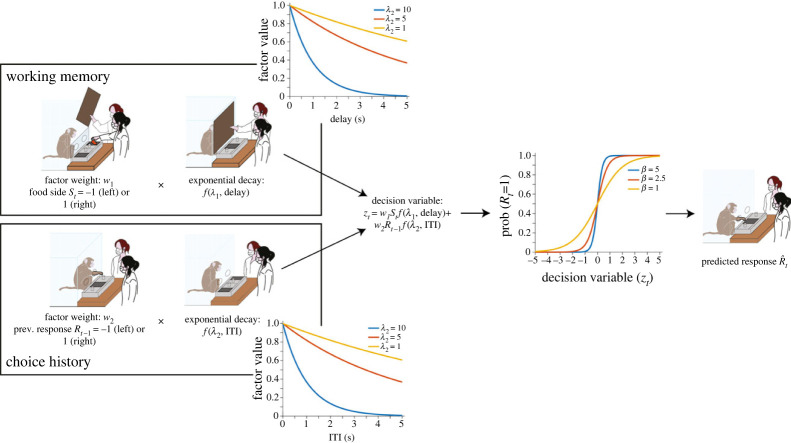
Model architecture. The model included a decaying working memory trace of the food location (top left, the plot shows the influence of decay rate: with higher decay rates, the influence of working memory diminishes faster) and a decaying trace of the previous choice (bottom left, the plot shows the influence of decay rate: with higher decay rates, the influence of the previous choice diminishes faster). The weighted sum of these influences is used to compute the probability of choosing one side or the other using a softmax function (the plot shows the influence of the inverse softmax temperature parameter: at higher values, the decision is more deterministic).

A computational model of decision making was fitted to the decision behaviour of each subject (chosen out of 11 candidate models based on model comparison; see electronic supplementary material). The model included two influences on the decision to choose the left or right side (figure 3). The first influence was a decaying working memory trace, *m*, of where the food was hidden. We modelled this as an exponential decay function according to the delay on trial *t*, *d_t_*:

$$m_{t}=w_{1}S_{t}e^{-\lambda_{1}d_{t}},$$

where *w*_1_ is the weight of this factor when the delay is 0, *S_t_* is the side that food was hidden on in trial *t* (left = −1, right = 1) and *λ*_1_ is the working memory decay rate. The second influence was that of the previous two choices, *p*. We assumed that the strength of this influence would also decrease with time [53], and we therefore also modelled this influence as an exponential decay function according to the ITI before trial *t*, *i_t_*:

$$p_{t}=w_{2}(R_{t-1}e^{-\lambda_{2}(i_{t}+d_{t})}+R_{t-2}e^{-\lambda_{2}(i_{t-1}+d_{t-1}+i_{t}+d_{t})}),$$

where *w*_2_ is the weight of this factor when the ITI and delay is 0, *R_t_*_−1_ is the response made on the previous trial (left = −1, right = 1) and *λ_2_* is the decay rate. Positive values of *w*_2_ therefore represent a tendency to repeat the previous choice, whereas negative values represent a tendency to suppress the previous choice, via different degrees of inhibitory control.

The decision variable, *z*, was computed as the sum of these two factors, *m* and *p*, with negative values of representing a tendency to choose the left side, and positive values a tendency to choose the right side. The decision variable was then transformed into the probability of choosing the right side on trial *t* using the softmax operator [54],

$$P(R_{t}=1)=\frac{1}{1+e^{-\beta z_{t}}}.$$

The inverse softmax temperature, *β*, determines the steepness of the softmax function, and thus how sensitive the choice probability is to *z*. We refer to this parameter as representing ‘choice stochasticity’, as it captures the balance between exploration and exploitation.

The model was fit separately to each subject's choice behaviour using maximum log-likelihood estimation in Matlab (v R2018a) to find the parameter values that optimize the likelihood that the model would produce the same choices. This process can get stuck in local maxima, a situation in which any small change to the current parameter values decreases the log-likelihood. To avoid this situation, the optimization can be run multiple times, each time with a different initial guess for each parameter value, taking the parameter values with the overall maximum log-likelihood. To ensure that a large portion of the space of possible parameter values was explored, a grid search over all parameter values was used to initialize model parameters before fitting [55]. The weight of the working memory factor, *w_1_*, was restricted to the range [0,1], and the weight of the choice history factor, *w_2_* was restricted to [−1,1] in order to detect both a tendency to inhibit or repeat the previous choice reflecting different degrees of inhibitory control. These ranges served to normalize the contributions of each factor to the decision variable. The decay rate parameters, *λ_1_* and *λ_2_*, and the inverse softmax temperature, *β*, were constrained to be in the range [0,10] to avoid asymptotic values in which further increases in value have no effect on behaviour.

### (f) Data analysis

Before analysis, we checked if any coded behaviours that occurred during either a trial or the ITI were affecting the ability of the model to predict the responses of the subjects. We found that locomotion during trials was the only behaviour negatively impacting the model's prediction accuracy, and therefore, we excluded all the trials in which locomotion occurred (see electronic supplementary material). We also removed trials in which the ITI was more than 2.5 s.d. above the mean, and we excluded subjects with less than 25 remaining trials (one mother-reared subject from both the original task at the first time-point and the randomized control version at the second time-point). We then checked if there was a difference in ITI duration between the two rearing groups, and we found no difference (see electronic supplementary material).

To compare performance on the original ‘A-not-B’ task between the two rearing groups over time, we computed three measures based on previous literature (e.g. [47,52]): (i) percentage of correct responses (i.e. proportion of correct trials out of all trials completed); (ii) cumulative score (the sum of successful change trial delays divided by the number of total trials completed); and (iii) maximum delay reached. These measures were computed for each subject at each assessment time-point. The percentage of correct responses was also computed for the randomized control version of the task completed at the second time-point. For percentage of correct responses, we conducted a generalized linear mixed model with group (mother-reared/peer-reared), time-point (3.5/5 years) and their interaction included as fixed effects, and subject, time-point and session as nested random intercepts. For the cumulative score and maximum delay, we conducted a linear mixed model with group, time-point and their interaction included as fixed effects, and subject-specific random intercepts. To compare the percentage of correct responses in the original task versus the randomized control version at the second time-point, we conducted a generalized linear mixed model with group, task (original/control) and their interactions included as fixed effects, and subject, task (original/control) and session as nested random intercepts.

The computational model was fitted separately for each subject at each assessment time-point, and for the two versions of the tasks. To compare the fitted model parameters between the two rearing groups across time, we used linear mixed models with group, time-point, and their interactions included as fixed effects, and subject-specific random intercepts. To compare the fitted parameters between the two rearing groups and tasks at the second time-point, we used linear mixed models with group (mother-reared/peer-reared), task (original/control) and their interactions included as fixed effects, and subject-specific random intercepts. To investigate whether the fitted model parameters were related to task performance, and thus to what extent the performance measures reflected involvement of working memory and inhibitory control, we used separate linear mixed models for each model parameter and performance measure in the original version across time. For percentage of correct responses, cumulative score and maximum delay, we computed linear mixed models with model parameter (i.e. *w*_1_, *λ*_1_, *w*_2_, *λ*_2_ or *β*), group (mother-reared/peer-reared), time-point (3.5/5 years) and group by time-point interaction included as fixed effects, and subject-specific random intercepts. We then used separate linear mixed models for each model parameter and performance measure across tasks at the second time-point. For the percentage of correct responses, we fit linear mixed models with model parameter (i.e. *w*_1_, *λ*_1_, *w*_2_, *λ*_2_ or *β*), group (mother-reared or peer-reared), task (original/control) and group by task interaction included as fixed effects, and subject-specific random intercepts.

R v. 4.0.5 [56] was used to conduct all analyses presented here (see electronic supplementary material for package information). *p*-values for fixed effects and interactions were obtained using Type III Wald *χ*^2^ tests for generalized linear models. To account for smaller sample sizes and normality violations, permutation tests (grouped by subjects) with 10 000 permutations were used to assess the significance of linear model factors and to follow-up significant interactions.

## 3. Results

### (a) Task performance

**Figure 4.**
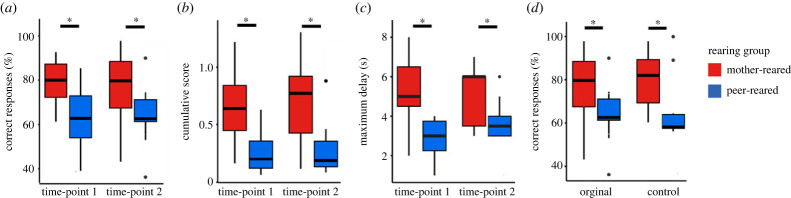
Task performance. The mother-reared group had a significantly higher percentage of correct responses (*a*), cumulative score (*b*) and maximum delay (*c*) in the original ‘A-not-B’ task than the peer-reared group at both time-points. Mother-reared animals also had a significantly higher percentage of correct responses in the randomized control task at the second time-point (*d*), and the performance of each group was not different between tasks.

We first sought to determine if task performance varied between groups and time-points by comparing each of the performance measures (percentage of correct responses, cumulative score and maximum delay) between groups and time-points for the original version of the ‘A-not-B’ task. A significant main effect of group was revealed for the percentage of correct responses [*χ*_1_ = 9.462; *p* < 0.01], with a higher performance for the mother-reared compared to peer-reared group (figure 4*a*). No significant main effect of time-point or group by time-point interaction was found. A significant main effect of group was also revealed for a cumulative score [*F*_1_ = 12.643, *p* < 0.01], with a higher score for mother-reared compared to peer-reared group (figure 4*b*). No significant effect of time-point or group by time-point interaction was found. Finally, a significant main effect of group was found for the maximum delay [*F*_1_ = 17.721, *p* < 0.001], with a higher delay for mother-reared compared to peer-reared group (figure 4*c*). Again, no significant effect of time-point or group by time-point interaction was revealed.

Having established that the mother-reared group had higher performance on the ‘A-not-B’ task than the peer-reared group at each time-point, we then compared their performance between the original ‘A-not-B’ task and the random control task at the second time-point. A significant main effect of group was found [*χ*_1_ = 6.323; *p* = 0.011], with a higher percentage of correct responses for the mother-reared compared to peer-reared group (figure 4*d*). No significant main effect of task or task by group interaction was revealed.

### (b) Computational model fits

**Figure 5.**
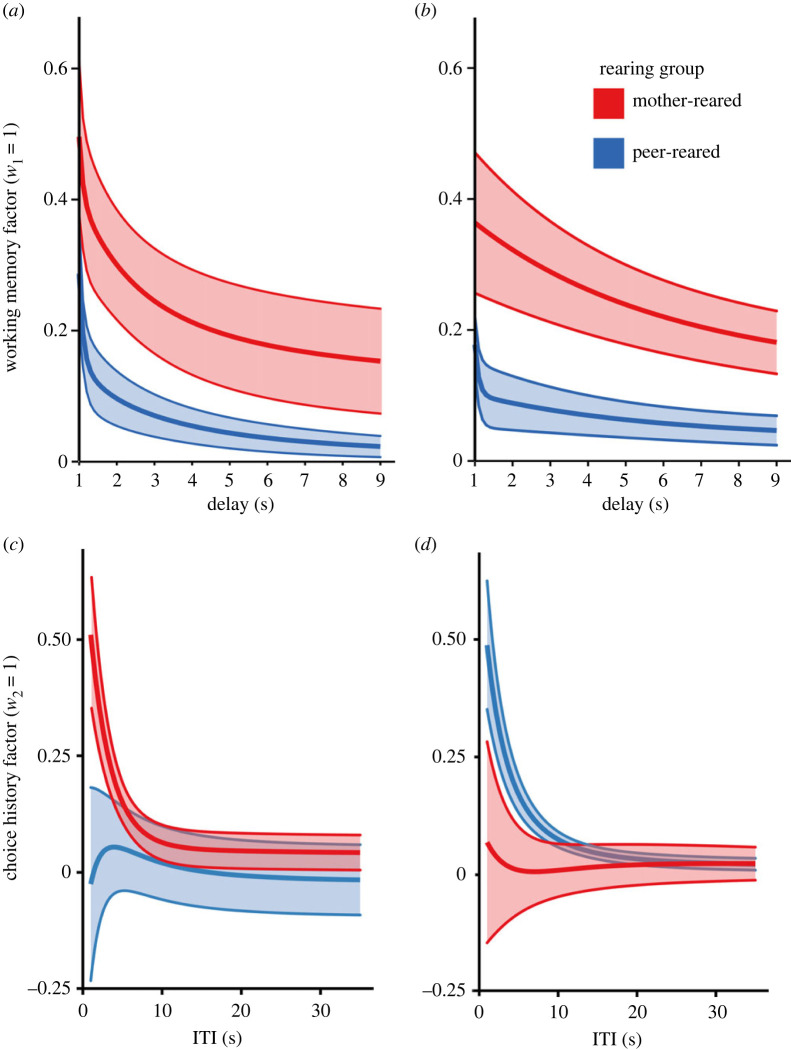
Fitted model parameters for the original ‘A-not-B’ task at both time-points. (*a*) Decay in the value of the working memory factor at the first time-point, given *λ*_1_, and fixing *w*_1_ = 1, for various delay durations. The solid lines represent the mean over subjects (red = mother-reared, blue = peer-reared), and the shaded areas represent the standard error. (*b*) As in (*a*), for the second time-point. (*c*) Decay in the value of the choice history factor rate at the first time-point, given *λ*_2_, and fixing *w*_2_ = 1, for various ITIs. (*d*) As in (*c*), for the second time-point.

In order to determine the cognitive mechanisms underlying the difference in performance between the two groups, we then compared the fitted model parameters between groups and time-points for the ‘A-not-B’ task. A significant main effect of group was revealed for working memory decay (*λ*_1_) [*F*_1_ = 7.008, *p* = 0.014], with the peer-reared group demonstrating a significantly faster rate of decay compared to the mother-reared group (figure 5*a,b*). There was no significant effect of time-point or group by time-point interaction and no significant differences in terms of the weight of the working memory factor (*w*_1_). There were no main effects of group or time-point on the weight of the choice history factor (*w*_2_), but a significant interaction between group and time-point was found [*F*_1_ = 7.159, *p* = 0.021], with *w*_2_ decreasing from time-point 1 to time-point 2 in mother-reared group and increasing in the peer-reared group. No significant main effects or interactions were found for choice history decay rate (*λ*_2_; figure 5*c*,*d*) nor choice stochasticity (*β*).

**Figure 6.**
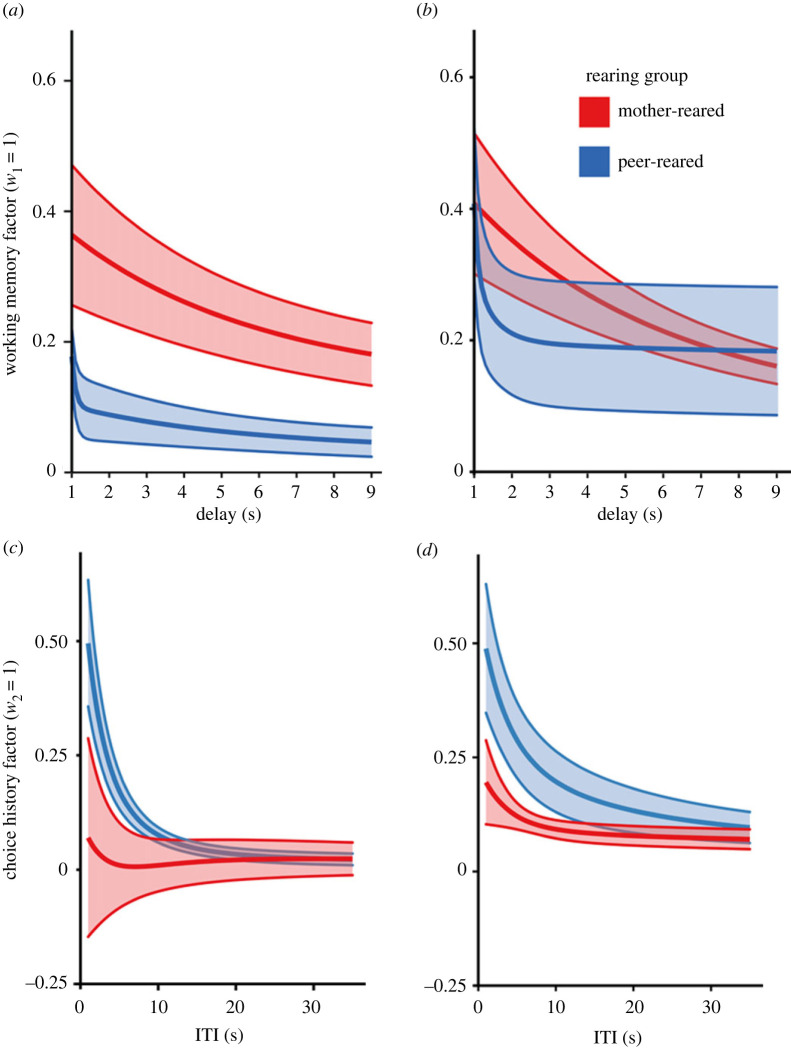
Fitted model parameters for the original and random control tasks at the second time-point. (*a*) Decay in the value of the working memory factor for the original ‘A-not-B’ task at the second time-point, given *λ*_1_, and fixing *w*_1_ = 1, for various delay durations. The solid lines represent the mean over subjects (red = mother-reared, blue = peer-reared), and the shaded areas represent s.e. (*b*) As in (*a*), for the random control task at the second time-point. (*c*) Decay in the value of the choice history factor rate for the original ‘A-not-B’ task at the second time-point, given *λ*_2_, and fixing *w*_2_ = 1, for various ITIs. (*d*) As in (*c*), for the random control task at second time-point.

We then aimed to establish if the mechanisms behind the difference in performance between groups were similar in both versions of the task by comparing their fitted model parameters between the original ‘A-not-B’ task and the random control task at the second time-point. For working memory decay rate (*λ*_1_), a significant main effect of group was revealed [*F*_1_ = 5.314, *p* = 0.022], with the peer-reared group demonstrating a significantly faster rate of decay compared to the mother-reared group (figure 6*a*,*b*). There was no significant effect of task, or a group by task interaction. Again, there were no significant differences in terms of the weight of the working memory factor (*w*_1_) (figure 6*a,b*). For choice history, a significant main effect of group was revealed, with a greater choice history factor weight (*w*_2_) found for the peer-reared compared to mother-reared group [*F*_1_ = 5.454, *p* = 0.031] (figure 6*c,d*). There was no significant effect of time-point or interaction effects. No significant main effects or interactions were found for decay rate of the choice history factor (*λ*_2_) (figure 6*c,d*). For choice stochasticity (*β*), there were no significant main effects or interactions.

### (c) Relationship between fitted model parameters and performance measures

Having shown that the groups vary similarly in terms of their performance in the original ‘A-not-B’ and random control tasks, and that the model predicts group differences in working memory decay and the influence of choice history, we then wanted to find out if these mechanisms were reflected by any of the performance measures calculated. We therefore related each fitted parameter to each performance measure for the original version of the task at both time-points. The weight of the working memory factor (*w*_1_) and the rate of working memory decay (*λ*_1_) predicted the percentage of correct responses [*w*_1_: *F*_1_ = 11.104, *p* < 0.01; *λ*_1_*: F*_1_ = 10.564, *p* < 0.01; *F*_1_ = 5.180, *p* = 0.048], with higher working memory factor weight and longer working memory decay rate predicting a higher percentage of correct responses. There was no relationship between the weight of the choice history factor (*w*_2_), the decay rate of the choice history factor (*λ*_2_) or choice stochasticity (*β*) and the percentage of correct responses. The cumulative score was predicted by the weight of the working memory factor (*w*_1_) [*F*_1_ = 4.576, *p* = 0.038], the rate of working memory decay (*λ*_1_) [*F*_1_ = 17.052, *p* < 0.01] and choice stochasticity (*β*) [*F*_1_ = 8.880, *p* < 0.01], with higher working memory factor weight, longer working memory decay rate and less choice stochasticity predicting higher cumulative scores. There was no relationship between the weight of the choice history factor (*w*_2_) or the decay rate of the choice history factor (*λ*_2_) with the cumulative score. Finally, the rate of working memory decay (*λ*_1_) [*F*_1_ = 19.487, *p* < 0.001] and choice stochasticity (*β*) [*F*_1_ = 9.941, *p* < 0.01] both predicted the maximum delay, with longer working memory decay rate and less choice stochasticity linked to higher maximum delay. Neither the weight of the working memory factor (*w*_1_), the weight of the choice history factor (*w*_2_), nor the decay rate of the choice history factor (*λ*_2_) predicted maximum delay.

We then repeated this analysis for the original ‘A-not-B’ task and the random control task at the second time-point, relating each fitted model parameter to the percentage of correct responses (the cumulative score and maximum delay metrics only apply to the original task structure). The weight of the working memory factor (*w*_1_) [*F*_1_ = 9.921, *p* < 0.01] and working memory decay rate (*λ_1_*) [*F*_1_ = 4.696, *p* = 0.046] both predicted the percentage of correct responses, with greater *w*_1_ and longer decay rate related to a higher percentage of correct responses. The weight of the choice history factor (*w*_2_), the decay rate of the choice history factor (*λ*_2_) and choice stochasticity (*β*) did not predict the percentage of correct responses.

## 4. Discussion

This study aimed to assess the longitudinal effects of early psychosocial deprivation on EF across adolescence and early maturity in rhesus macaques, using an ‘A-not-B’ task. We found that early psychosocial deprivation had a negative effect on task performance, with the peer-reared group performing worse than the mother-reared group at both assessment time-points. Furthermore, fitting a computational model of decision making enabled us to identify the mechanistic processes likely to be contributing to performance on the task, with results suggesting that psychosocial deprivation has long-term effects on both working memory and inhibitory control components of EF. In addition to offering important insights into the longitudinal effects of early deprivation on EF, findings also support a protracted developmental trajectory of inhibitory control in macaques that extends into adulthood. These results indicate that the mechanisms implicated in the development of specific EF components are similar in macaques and humans, with specific EF components having different developmental timelines. Our study therefore also provides support for the use of macaque models to investigate the psychological and neural mechanisms through which EF develops more generally.

Our findings provide clear evidence for the potential long-term negative impact of early psychosocial deprivation on EF in macaques. Mother-reared animals performed better on the ‘A-not-B’ task than peer-reared animals across adolescence (time-point 1: 3.5 years) and early adulthood (time-point 2: 5 years), which is in line with previous findings suggesting that early psychosocial deprivation effects on childhood EF in humans can persist into adulthood [21]. Analysis of the fitted model parameters revealed differences between the groups in terms of working memory, with peer-reared animals having a faster rate of working memory decay across time. A difference in terms of inhibitory control (i.e. the influence of choice history) was also found, with peer-reared subjects having a greater tendency to repeat, rather than inhibit, their previous choice in early adulthood. Notably, results from the modified random version of the task, used as a control at the second time-point (5 years), indicate that these group differences were not simply due to learning the structure of the original ‘A-not-B’ task. These effects on specific components of EF are in keeping with evidence that adversity in the form of early deprivation may have a particularly severe impact on working memory and inhibitory control in humans (e.g. [22]).

Notably, analysis of the fitted model parameters suggests that early psychosocial deprivation was linked to poor working memory in adolescence and early adulthood, but to weaker inhibitory control in early adulthood only. This could be due to several different factors. One possibility is that the performance directly impacted the ability of the task to correctly assess inhibitory control. According to Diamond & Goldman-Rakic [45], the ‘B’ error (i.e. an incorrect response after switching the hiding position) occurs only when performance is accurate enough. This would mean that inhibitory control is only assessed correctly when accuracy is relatively high. Many studies use a criterion for subject inclusion where the participant has to be successful in at least one ‘B’ trial to be included in analysis (e.g. [47]). We also used this criterion, and the model successfully predicted the same proportion of responses for both rearing groups despite their differences in performance (see electronic supplementary material). It is therefore unlikely that the differences in the fitted model parameters would be due to performance differences. Another possibility is that working memory and inhibitory control follow different developmental trajectories. For example, evidence shows that inhibitory control starts to decline later (around 35 years) than working memory in human adults [4], and in macaques, evidence suggests that working memory is mature even at pre-puberty whereas inhibitory control is not [57]. However, a lack of longitudinal studies that focus on both working memory and inhibitory control does not allow any clear conclusion to be made. Based on our findings, we propose that while early psychosocial deprivation may impact both aspects of EF, their developmental trajectories after exposure likely differ. Peer-reared animals could have accelerated development of inhibitory control compared to mother-reared, then during adolescence, they are less flexible and do not improve. On the contrary, working memory may be impaired or accelerated at an earlier stage of development, with a protracted and stable negative effect of early psychosocial deprivation then seen across the transition from adolescence to adulthood. These differing trajectories could be explained by distinct underlying brain networks that are differentially impacted by early psychosocial deprivation. Confirming this hypothesis now requires more studies assessing neural measures development and actual link to behaviour across various stages of development.

Importantly, the inhibitory control parameters of the computational model (i.e. the influence of choice history), did not predict any of the performance measures, including the cumulative score and maximum delay, which are thought to account for both inhibitory control and working memory EF components. On the contrary, the working memory parameters predicted all performance measures. This suggests that the performance measures used in previous research mainly capture the influence of working memory on behaviour. However, using the computational model, it was possible to assess the contribution of both working memory and inhibitory control separately using an ‘A-not-B’ task. These findings are in line with the recent suggestion that proper assessment of inhibitory control in the first years of life is difficult using ‘A-not-B’ and similar tasks because of the high demands of working memory necessary to complete such tasks [47,50]. Fitting of computational models could thus help us to understand if EF follows an unitary construct in infancy or if different components can already be distinguished at a very early stage of development still using this task [3], and how these factors can lead to individual differences related to both positive and negative outcomes. Our results suggest that the ‘A-not-B’ task does correctly assess inhibitory control, but this EF component is not reflected by the measures of performance used in the previous literature.

There are a few limitations to the present study that should be acknowledged. First, due to the sample size, it is difficult to account for inter-individual variability within the two groups and the results may thus not be generalizable. Second, we did not have any time-points pre-puberty, which would be necessary to fully examine developmental trajectories of EF development after exposure to early psychosocial deprivation. Third, we only had the control version of the task at the second assessment time-point (5 years). It will be important in future research to replicate these results with careful controls in larger samples and at time-points covering multiple key transition periods in development.

This study also has several strengths. First, we assessed the effect of early psychosocial deprivation on EF development at two time-points covering a key transition period (i.e. adolescence into early adulthood). Second, our sample included two groups of macaques exposed to different early social and highly controlled environments. Third, all assessments were conducted with the monkeys remaining in their home enclosure without the addition of invasive and stressful procedures, which also makes results more comparable to those from human studies [49]. Finally, the use of a computational model enabled the distinction between specific aspects of EF. This approach could be of great use in future longitudinal assessment of EF, especially when including a wide range of key developmental transition points.

To conclude, results from this study demonstrate that early psychosocial deprivation is associated with long-term effects on EF, which are apparent in adolescence and persist into adulthood. The use of a computational model of decision making to disentangle mechanisms underlying performance on the ‘A-not-B’ task provides an alternative way to analyse the data from such tasks and may be of specific interest for developmental research, which is often defined by a lack of precise measurement tools. In the future, computational approaches such as that used here can easily be adapted to other EF tasks, with the results then more easily linked to specific aspects of neural development and related cognitive mechanisms than classic behavioural measures alone. Such research involving assessment of brain development is essential for clarifying how early deprivation effects EF over the lifespan and identifying the factors that confer risk for or resilience against poor developmental outcomes. This will be critical for design of more effective treatments and interventions that target individuals more at risk for developing impairments in specific aspects of EF and associated difficulties after exposure to early psychosocial deprivation.

## Data Availability

The data are provided in the electronic supplementary material [58].
